# Portrait Segmentation Using Ensemble of Heterogeneous Deep-Learning Models

**DOI:** 10.3390/e23020197

**Published:** 2021-02-05

**Authors:** Yong-Woon Kim, Yung-Cheol Byun, Addapalli V. N. Krishna

**Affiliations:** 1 Centre for Digital Innovation, CHRIST (Deemed to be University), Bangalore, Karnataka 560029, India; 2 Department of Computer Engineering, Jeju National University, 102 Jejudaehak-ro, Jeju-si 63243, Korea; 3 Department of Computer Science and Engineering, CHRIST (Deemed to be University), Bangalore, Karnataka 560029, India; adapalli.krishna@christuniversity.in

**Keywords:** portrait segmentation, deep learning, ensemble, simple soft voting, weighted soft voting, stacking, efficiency

## Abstract

Image segmentation plays a central role in a broad range of applications, such as medical image analysis, autonomous vehicles, video surveillance and augmented reality. Portrait segmentation, which is a subset of semantic image segmentation, is widely used as a preprocessing step in multiple applications such as security systems, entertainment applications, video conferences, etc. A substantial amount of deep learning-based portrait segmentation approaches have been developed, since the performance and accuracy of semantic image segmentation have improved significantly due to the recent introduction of deep learning technology. However, these approaches are limited to a single portrait segmentation model. In this paper, we propose a novel approach using an ensemble method by combining multiple heterogeneous deep-learning based portrait segmentation models to improve the segmentation performance. The Two-Models ensemble and Three-Models ensemble, using a simple soft voting method and weighted soft voting method, were experimented. Intersection over Union (IoU) metric, IoU standard deviation and false prediction rate were used to evaluate the performance. Cost efficiency was calculated to analyze the efficiency of segmentation. The experiment results show that the proposed ensemble approach can perform with higher accuracy and lower errors than single deep-learning-based portrait segmentation models. The results also show that the ensemble of deep-learning models typically increases the use of memory and computing power, although it also shows that the ensemble of deep-learning models can perform more efficiently than a single model with higher accuracy using less memory and less computing power.

## 1. Introduction

Image segmentation has been one of the most challenging problems in image processing and computer vision for the last three decades. It is different from image classification, as an image classification algorithm has to classify the object which has a particular label, but an ideal image segmentation algorithm has to segment even the unknown objects [1]. Good image segmentation is expected to have uniform and homogeneous segmented regions, but achieving a desired segmented image is a challenging task [2]. Numerous image segmentation algorithms have been developed and reported in the literature. The traditional image segmentation algorithms are generally based on clustering technology, and the Markov model is one of the most well-known approaches. In general, the traditional image segmentation algorithms based on digital image processing and mathematical morphology are susceptible to noise, and require a lot of interaction between a human and computer for accurate segmentation [3,4,5,6,7,8,9,10,11,12,13]. Amongst image segmentation algorithms, Deep Learning (DL)-based image segmentation has created a new generation of image segmentation models, and surpassed traditional image segmentation methods in many aspects [14,15]. DL-based image segmentation aims to predict a category label for every image pixel, which is an important yet challenging task [16]. Ensemble methods can be used for improving prediction performance, and the idea of ensemble method is to build an effective model by integrating multiple models [17]. Model averaging is a general strategy amongst many ensemble methods in machine learning, and its idea is to train several different models separately, and then all the models vote on the output of each model. It is known that neural networks provide a wide variety of solutions, and can benefit from model averaging, even if all of the models are trained on the same dataset [18].

Integrating multiple models to enhance the prediction performance has been studied and analyzed by several researchers in machine learning: Breiman explained a method to improve the performance accuracy by combining several models [19]; Warfield et al. presented an algorithm for combining and estimating the segmentation performance [20]; Rohlfing et al. introduced a new way to combine multiple segmentation and enhance performance [21]; Hansen and Salamon suggested that an ensemble of similar functioning and configured networks increased the predictive performance of the individual network [22]; Singh et al. specified that ensemble segmentation had resulted in better performance than individual segmentation [23]; Yan Zou et al. introduced a rapid ensemble learning by combining deep convolution networks and random forests to reduce learning time using limited training data [24]; and Andrew Holliday et al. suggested a model compression technique to speed up DL segmentation using an ensemble. The authors combined the strengths of different architectures, while maintaining the real-time performance of segmentation [25]. Ishan Nigam et al. proposed an ensemble knowledge transfer to improve aerial segmentation. The authors trained multiple models by progressive fine-tuning and combined the collections of models to improve performance [26]. D. Marmanis et al. suggested an ensemble approach using Fully Convolution Network (FCN) models for the semantic segmentation of aerial images. It has been shown in this work that ensembles of several networks achieve excellent results [27]. Machine Learning contests are usually won by methods using model averaging, for example the Netflix Grand Prize [28]. Y. W. Kim et al. introduced an ensemble approach by combining three DL-based portrait segmentation models, and showed significant improvement in the segmentation accuracy [29].

Semantic image segmentation plays a central role in a broad range of applications such as medical image analysis, autonomous vehicles, video surveillance and Augmented Reality (AR) [14,30]. Portrait segmentation, which is a subset of semantic image segmentation, is generally used to segment a human’s upper body in an image, but may not be limited to the upper body. The use of portrait segmentation technology is becoming more and more popular due to the popularity of “selfies” (self-portrait photographs). Portrait segmentation is widely used as a pre-processing step in multiple applications such as security system, entertainment application, video conference, AR, etc. In portrait segmentation, the precise segmentation of the human body is crucial but challenging [31]. A substantial amount of DL-based portrait segmentation approaches have been developed since the performance and accuracy of semantic image segmentation have improved significantly due to the recently introduced DL technology [32,33,34,35,36,37,38,39,40,41]. However, studies related to these approaches are focused on a single portrait segmentation model.

In this paper, we propose a novel approach using ensemble method by combining multiple heterogeneous DL-based portrait segmentation models, and the experiment results show that the proposed approach can produce improved performance over single DL-based portrait segmentation models. The major contributions of this research are as follows:

(1) A novel approach of combining multiple heterogeneous DL-based portrait segmentation models was introduced. According to the best of our knowledge, there are no reported works on portrait segmentation using an ensemble of multiple heterogeneous DL models.
(2) The efficiency rate of memory and computing power was measured to evaluate the efficiency of portrait segmentation models. We attempted to measure cost efficiency of five state-of-the-art DL-based portrait segmentation models, and proposed an ensemble approach. As per the best of our information and relevant survey, we have not found any reported works measuring cost efficiency for DL-based portrait segmentation models.
(3) Intersection over Union (IoU), IoU standard deviation, False Negative Rate (FNR), False Discovery Rate (FDR), FNR + FDR and |FNR-FDR| were used to measure the accuracy, variance and bias error of segmentation results. We have used IoU standard deviation to measure the variance error of experimented models, FNR + FDR to measure the bias error and |FNR-FDR| to measure the balance of bias error. These metrics are a new approach to evaluate the performance of DL-based portrait segmentation models.

In addition to these major contributions, the minor contributions of our research work are presented below:

(4) Six state-of-the-art DL-based portrait segmentation models were experimented on and compared.
(5) The Simple Soft Voting method and Weighted Soft Voting method were used to make an ensemble of individual portrait segmentation models.
(6) A simple and efficient way to combine the output results of individual portrait segmentation models was introduced.
(7) A quantitative experiment was used to evaluate the performance of portrait segmentation models.

Section 2 gives a brief introduction about the portrait segmentation and soft voting method. Section 3 explains portrait segmentation models used in the experiment, the concept of an ensemble, approach to combine portrait segmentation models, evaluation metrics and experiment environment. Section 4 presents the results of the experiment, followed by the brief discussion of the results in Section 5. The summary of experiment and contribution of this work are covered in Section 6.

## 2. Background

### 2.1. Portrait Segmentation

In general, segmentation subdivides an image into its constituent regions or objects. The application problem defines the level to which the subdivision is supposed to be carried out, i.e., the segmentation should stop once the object of interest is segmented. The segmentation accuracy determines the success or failure of the application problem. Acknowledging the importance of segmentation applications in the real world specifies the necessity for obtaining highly precise and accurate segmented images. Portrait segmentation in general can be referred as a process of segmenting a person in an image from its background. There are many applications using a portrait segmentation technology. Some popular camera apps can change the background of a selfie photo automatically with one-click. A portrait segmentation is used for online video conferencing, as well as entertainment. Many organizations are using online video conferencing solutions, as many people are working from home since the COVID-19 pandemic. Portrait segmentation technology is used to hide the user’s background for privacy, security and aesthetic reasons, while users are attending the online video conferencing. In addition to these, the portrait segmentation technology can be used for content-based image retrieval. For example, a segmented human body in photos can be stored in a database. When a user enters a query to find all photos with similar people, the human body of the entered photo can be segmented and passed to the database to find similar people in the database.

Recently, a substantial amount of DL-based portrait segmentation networks was developed in the literature. Song-Hai Zhang et al. proposed a novel semantic segmentation network called PortraitNet, which was specifically designed for real-time portrait segmentation on mobile devices with limited computational power, and their experimental results demonstrate both high accuracy and efficiency [33]. Hyojin Park et al. introduced SINet, which executes well on mobile devices with 100.6 frames per second, and has a high accuracy of 95.29% [34]. FCN was proposed by Jonathan Long et al., and adapts the contemporary classification networks into fully convolutional networks and transfers their learned representations by fine-tuning to the segmentation task, achieving a state-of-the-art performance [35]. Mark Sandler et al. proposed a new mobile architecture, MobileNetV2, which improves the performance of mobile models on multiple tasks and benchmarks [36]. Its main contribution is a novel layer module. Andrew Howard et al. presents the next generation of MobileNets called MobileNetV3 in their work, based on a combination of complementary search techniques as well as novel architecture design [37]. Jimei Yang et al. introduced a DL algorithm for Contour Detection with a Fully Convolutional Encoder-Decoder Network (CEDN) [38]. This Fully Convolutional Encoder-Decoder was developed, inspired from a fully convolutional network and unpooling layers from deconvolutional network, and it focuses on detecting higher level object contours. Authors compared it with traditional methods and observed that proposed DL-based contour detections outperformed traditional methods and other DL-based approaches. Xianzhi et al. proposed a boundary-sensitive network for portrait segmentation, and authors showed the DL-based portrait segmentation outperformed the graph cut method [31]. Hyojin Park et al. introduced a new extremely lightweight portrait segmentation model named ExtremeC3Net, which even with far fewer parameters, produced competitive segmentation accuracy with PortraitNet [39]. DeepLabV3 Network was introduced by Liang-Chieh Chen et al., which significantly improves over their previous DeepLab versions without DenseCRF post-processing, and attains comparable performance with other state-of-art models [40]. Xi Chen et al. proposed the Boundary-Aware network (BANet), which is a lightweight network architecture that focuses on extracting detailed information in the boundary area to produce a high-quality segmented image in less time [41].

**Figure 1:**
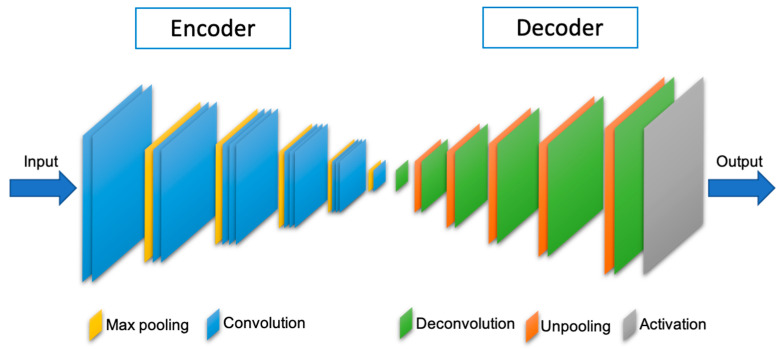
Structure of Fully Convolutional Encoder-Decoder Network (CEDN) [38].

A segmentation model learns a desired task from the given inputs to produce the expected output. In this paper, we combine the outputs of DL-based image segmentation models. These models use the Convolution Neural Network (CNN) DL algorithm. CNN has led to the development of state-of-the-art segmentation models, among which six portrait segmentation models are used in this paper. The FCN model owes its name to its architecture; this model uses a novel method to obtain a better upsampled feature map. The MobileNetV2 model has used a unique architecture to improve the performance on small devices. The other portrait segmentation models used in this paper have their architectures built based on the Encoder-Decoder architecture. The pooling layer in encoder module produces low-resolution feature map containing high-level information, and the decoder module aims to produce high-resolution segmentation output from the high-level information received from the encoder. Figure 1 shows the simple structure of the Encoder-Decoder model in general, for portrait segmentation.

### 2.2. Soft Voting Ensemble

In contrast to ordinary learning approaches, which try to construct one learner from training data, ensemble methods try to construct a set of learners and combine them. Generally, an ensemble is constructed in two steps, i.e., generating the base learners and then combining them. To get good ensemble, it is generally believed that the base learners should be as accurate and diverse as possible. There are many ensemble learning algorithms. Averaging and Voting are the most popular and fundamental ensemble methods for numeric and nominal outputs [42]. Considering classification as an example, the working of voting can be explained as, supposing a set of T individual classifiers {***h***_1_, …, ***h***_***T***_} are given where ***h*** indicates the classifiers. The task is to combine set of classifiers to predict the class label from a set of ***l*** possible class labels {***c***_1_, …, ***c***_l_}, where ***c*** is the class label. There are many types of voting, such as Majority voting, Plurality voting, Weighted voting, Soft voting, etc. Soft voting is used for individual classifiers which produce class probability outputs. In this method, the individual classifier ***h***_***i***_ outputs a ***l***-dimensional vector (***h***^1^(***x***), …, ***h***^***l***^(***x***))^***T***^ for instance of ***x***, where ***h***^***j***^(***x***) ∈ [0, 1] i.e., ***h***^***j***^(***x***) which is the output of classifier ***h***_***i***_ for the class label ***c***_***j***_, can be regarded as an estimate of the posterior probability. Soft voting is further classified into Simple Soft Voting and Weighted Soft Voting. In simple soft voting the individual classifiers are treated equally, and it generates the combined output by simply averaging all the individual outputs. The final output for class ***c_j_*** is given in Equation (1) below [42]:

(1) $$H^{j}\left(x\right)=\;\frac{1}{T}\overset{T}{\underset{i=1}{\sum }}h_{i}^{j}\left(x\right)$$

In weighted soft voting, the individual outputs are combined with different weights. The weighted soft voting method takes any of the following three forms:

- A class-specific weight is assigned to each classifier, and the combined output for class $c_{j}$ is given in Equation (2) below

(2) $$H^{j}\left(x\right)=\;\overset{T}{\underset{i=1}{\sum }}\;w_{i}h_{i}^{j}\left(x\right)$$

where $w_{i}$ is the weight assigned to the classifier $h_{i}$.

- A class-specific weight is assigned to each classifier per class, and the combined output for class $c_{j}$ is given in Equation (3) below

(3) $$H^{j}\left(x\right)=\;\overset{T}{\underset{i=1}{\sum }}w_{i}^{j}h_{i}^{j}\left(x\right)$$

where $w_{i}^{j}$ is the weight assigned to the classifier $h_{i}$ for the class $c_{j}$.

- A weight is applied to each example of each class for each classifier, and the combined output of $c_{j}$ is given in Equation (4) below

(4) $$H^{j}\left(x\right)=\overset{T}{\underset{i=1}{\sum }}\overset{m}{\underset{k=1}{\sum }}w_{ik}^{j}h_{i}^{j}\left(x\right)$$

where $w_{ik}^{j}$ is the weight of the instance $x_{k}$ of the class $c_{j}\;$for the classifier $h_{i}$.

Equation (4) is not often used in real practice, since it may involve a large number of weight coefficients. The weights $w_{i}$’s are usually assumed to be constrained, as given in Equation (5) below:

(5) $$w_{i}\geq 0\;\mathrm{and}\;\overset{T}{\underset{i=1}{\sum }}\;w_{i}=1$$

In this paper, simple soft voting using Equation (1) and weighted soft voting using Equation (2) are used to make an ensemble of the individual portrait segmentation models, as they are simple and efficient approaches to obtain effective results.

## 3. Materials and Methods

### 3.1. Experimented Portrait Segmentation Models

In this paper, we used PortraitNet (PN), SINet (SN), FCN, MobileNetV2 (MNV2), MobileNetV3 (MNV3) and the CEDN for our experiment. Table 1 shows the model names, the dataset used for training the model, the input image resolution of the model and the dataset used for testing the model.

The AISegment dataset [43], which is publicly available for portrait segmentation training and testing, was used to train the CEDN model. The Custom dataset consists of 18,698 portrait images which are publicly available on websites, and was used by the MobileNetV2 and MobileNetV3 pre-trained models. The EG1800 dataset [44] consists of 1447 publicly available images for training and 289 images for validation. In this paper, 270 images from the validation set of EG1800 dataset and 298 validation portrait image datasets collected from publicly available websites (we call it the “CDI” dataset) were used for the experiment.

### 3.2. Ensemble of Portrait Segmentation Models

**Figure 2:**
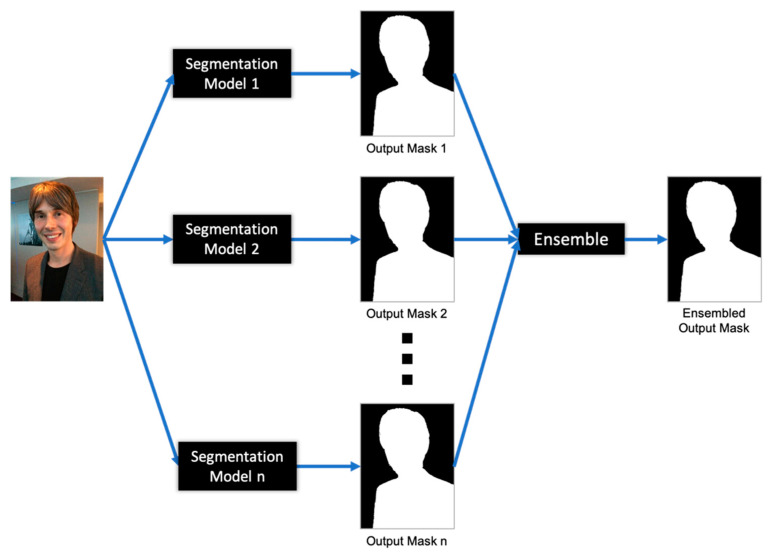
Ensemble of *n* portrait segmentation models.

In machine learning, ensemble models are developed by combining the prediction of multiple individual models to improve the overall predictions. The ensemble model can be created either by combining multiple modelling algorithms or using different training datasets. The main purpose of ensemble method is to reduce the generalization error in the machine learning algorithms. In this paper, we propose the ensemble approach of *n* portrait segmentation models to enhance the accuracy performance of the single models, where *n* indicates the number of single models combined at a time. Figure 2 shows the structure of ensemble approach of *n* portrait segmentation models. Consider a set of ***T*** number of portrait segmentation models i.e., {***L***_1_, ***L***_2_, ***L***_3_, …, ***L***_***T***_}, where ***L***_***i***_ are individual portrait segmentation models (First-Level learners). The input images from the EG1800 + CDI datasets are fed to the individual models, and the segmented output masks are obtained and stored. These individual segmented output masks are combined to enhance final outputs. For the ensemble of the outputs of individual models, the simple soft voting method and weighted soft voting method are used in this research. The idea of the ensemble is to learn from the outputs of individual models, and collectively produce a better segmented result.

**Figure 3:**
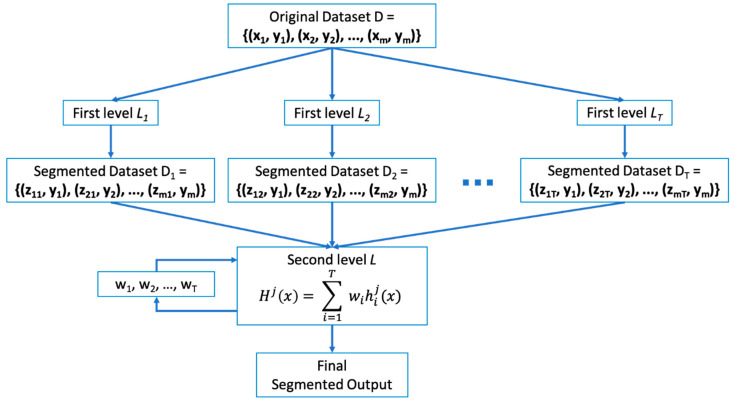
Diagram of stacking ensemble.

In this paper, weighted soft voting method is used as a Meta-Classifier of stacking ensemble. Stacking is the process of training individual classifiers called First-Level learners and combiners called Second-Level learners, or Meta-Learners that combine them [19,45,46]. The basic idea of stacking ensembles is to train First-Level learners using the original dataset, and the classification results generated by these learners will be used as a new training dataset for Second-Level learner. Here, the labels of the original dataset are still used as the labels of the new training dataset. Figure 3 shows the diagram of stacking ensemble, where D, the original dataset {(**x_1_, y_1_**), (**x_2_, y_2_**, …, (**x_m_, y_m_**)}, ***L***_1_, …, ***L_T_*** are First-Level learners, and ***L*** is the Second-Level learner, where *T* is the number of learners and *m* is the number of datasets. Although heterogeneous learning algorithms are often used as First-Level learners, it is also possible to use homogeneous learning algorithms. In this paper, segmentation models of Table 1 are used as First-Level learners and weighted soft voting is used for Second-Level learners. First level learners ***L***_1_, …, ***L_T_*** are executed using individual segmentation models. The segmentation results from these First-Level learners are collected, and the collected new dataset and original ground truth are fed to the Second-Level learner to train optimal weight values for weighted soft voting. For simple soft voting, weight values are not trained as the results of First-Level learners are treated equally. The Second-Level learner learns weight values for ensemble from the results of First-Level learners (D_1_, D_2_, …, D_*T*_).

### 3.3. Concept of Combining Segmented Outputs

**Figure 4:**
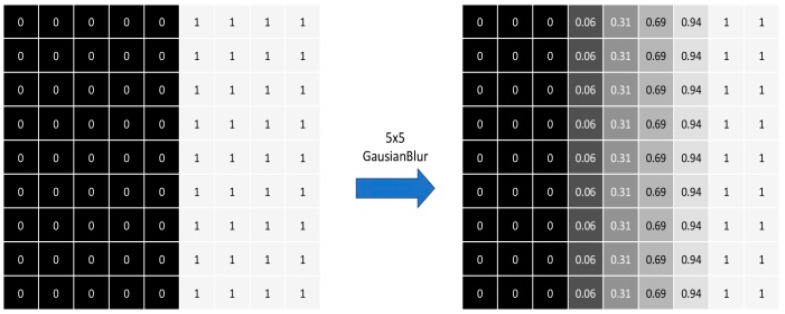
Converting binary value to probability value.

In this paper, simple soft voting and weighted soft voting as a Meta-Classifier in a stacking ensemble were used to combine the segmented output masks of individual models. In the simple soft voting method using Equation (1), the weights of outputs of individual models are treated equally. In weighted soft voting using Equation (2), the weights of outputs of individual models are not equal, and are trained from the result of First-Level classifiers in the stacking ensemble to find a respective weight ratio. The segmented output mask generated from individual models have a binary value, i.e., 1 or 0. This binary value was converted into probability value by applying the Gaussian Blur function. Gaussian Blur is commonly used in image processing to smoothen and reduce the noise in the image. Gaussian Blur uses a non-uniform low pass filter that preserves the low spatial frequency, and reduces the noise and negligible details in the image. It belongs to linear smoothing filters class with weights according to the shape of a Gaussian function [47]. The 2-Dimensional Gaussian filter is defined in Equation (6) below [48]

(6) $$G\left(x,y\right)=\;\frac{1}{2\pi \sigma^{2}}\;e^{\frac{-\left(x^{2}+y^{2}\right)}{2\sigma }}$$

where *G* is the Gaussian mask at the location with coordinates *x* and *y*, and *σ* is the parameter which defines the standard deviation of the Gaussian. The value of *σ* is directly proportional to the image smoothing effect. In this paper, Gaussian Blur is used to convert the binary image mask into the probability distribution, which indicates how much the pixel is close to the segmented human region. Figure 4 shows the effect of applying Gaussian Blur to a binary image mask. The left metrics shows 9 × 9 pixels divided into two values, 1 or 0. If the pixel value is 1, then it belongs to the segmented foreground region. If the pixel value is 0, then it belongs to the background region. The right metrics shows that the binary pixel values are converted into probability values after applying Gaussian Blur using a 5 × 5 kernel. If the pixel value is closer to 1, then it has higher probability to belong to the segmented foreground region. Converting the binary pixel value into the probability value is also useful to calculate Equations (1) and (2).

### 3.4. Performance Measurement

In this paper, the IoU metric was used for performance measurement. The IoU is a metric used to measure the accuracy of a classifier. When *A* is a segmented image and *B* is a ground truth image, then IoU is defined as the Equation (7) below

(7) $$\mathrm{IoU}=\;\frac{A\cap^{\;}B}{A\cup^{\;}B}$$

where *A* ∩ *B* is intersection of *A* and *B*, and *A* ∪ *B* is the union of *A* and *B*. In this paper, we used IoU standard deviation to measure the variance of prediction. FNR and FDR were calculated to validate the accuracy enhancement. FNR measures the false rate of the segmentation area, which is smaller than the ground truth. FDR measures the false rate of the segmentation area, which is larger than the ground truth. FNR and FDR are given as in Equations (8) and (9) below

(8) $$\mathrm{FNR}=\frac{\mathrm{FN}}{\;\mathrm{FN}+\mathrm{TP}\;}$$

(9) $$\mathrm{FDR}=\frac{\mathrm{FP}}{\;\mathrm{FP}+\mathrm{TP}\;}$$

Where TP is True Positive, FP is False Positive and FN is False Negative values of the segmented image. It is known that the ensemble of multiple classifiers can reduce the bias error of classifiers. In this paper, we used FDR + FNR to measure the bias error, as the bias error indicates the amount of difference between the prediction and true values.

To measure cost efficiency of models, the Memory Efficiency Ratio (MER) and Computing Efficiency Ratio (CER) are used. In general, the efficiency ratio indicates the costs as a percentage of gain (costs/gain). MER measures the memory efficiency of a model, which indicates the required memory size to gain certain accuracy. When P is parameter size of a model and IoU is accuracy metric of a model given by Equation (7), then MER is defined as Equation (10) below:

(10) $$\mathrm{MER}=\frac{P}{\;\;\;\;\mathrm{IoU}\;\;\;\;}$$

CER measures the computing power efficiency of the model, which indicates the required computing power to gain certain accuracy. When C is the Floating Point Operations (FLOPs) of a model and IoU is accuracy metric of a model given by Equation (7), then CER is defined as Equation (11) below:

(11) $$\mathrm{CER}=\frac{C}{\;\mathrm{IoU}\;}$$

### 3.5. Experiment Environment

In this paper, two groups of five state-of-the-art portrait segmentation models were used for the experiment. One group is used to evaluate IoU value, IoU standard deviation and false prediction rate, while another group is used to evaluate cost efficiency. For the experiment, the Two-Models ensemble and Three-Models ensemble were used to demonstrate the proposed ensemble approach. All possible two and three combinations of five single models were experimented for accuracy and cost efficiency measurements. The outputs of single models were combined using simple soft voting and weighted soft voting as a Meta-Classifier in a stacking ensemble. Equation (1) for simple soft voting and Equation (2) for weighted soft voting were used for the ensemble of single models. The validation images of EG1800 and CDI dataset were fed to the single models, and then the segmented output masks from these single models were combined using ensemble methods. A total of 568 portrait images of the EG1800 + CDI dataset were used to evaluate the accuracy and cost efficiency of individual portrait segmentation models and proposed ensemble models. The experiment results of Two-Models and Three-Model ensemble were compared with the result of single models and analysed. This experiment was conducted on an Ubuntu 18.4.4 LTS operating system. All the training and testing was performed using GeForce GTX 1080 GPU.

## 4. Results

In this section, the experiment’s result and its analysis of single models, Two-Models ensemble and Three-Models ensemble, are presented. IoU, IoU standard deviation, FNR and FDR are used to measure the accuracy of single models and suggested ensemble models.

### 4.1. Result of Individual Portrait Segmentation Models

Table 2 shows the experiment results of five single models. The best performed results are highlighted in bold. The MNV2 model shows 96.2082% of IoU, and is the highest accuracy among the experimented single models. The FCN model shows the lowest accuracy, with 95.2268% of IoU. The false prediction rate (FNR + FDR) shows a lower value, when the model has a higher IoU value.

### 4.2. Result of Two-Models Ensemble

In Table 3 and Table 4, all possible combination of Two-Models ensemble and Three-Models ensemble of five single models were experimented on using simple soft voting and weighted soft voting as a Meta-Classifier in a stacking ensemble. The first column shows the model combination; the other columns show IoU, IoU standard deviation and false prediction rate (FNR + FDR). Table 3 shows the experiment result of Two-Models ensemble. The best results of the experiment are highlighted in the table. In the experiment, the FCN + PN ensemble shows the highest IoU, and the lowest false prediction rate for both simple soft voting and weighted soft voting. FCN + MNV2 ensemble also shows good results similar to the FCN + PN ensemble. It is also observed that the weighted soft voting as a Meta-Classifier in a stacking ensemble produces a higher IoU value than the simple soft voting, i.e., the accuracy of ensemble models using the weighted soft voting is better than simple soft voting in a same model combination.

### 4.3. Result of Three-Models Ensemble

Table 4 shows the result of Three-Models ensemble. The best results of the experiment are highlighted in the table. The FCN + MNV2 + SN ensemble shows the highest IoU and the lowest false prediction rate in both simple soft voting and weighted soft voting. Like the result of Two-Models ensemble, it is observed that the weighted soft voting produces higher accuracy than the simple soft voting.

### 4.4. IoU Comparison

**Figure 5:**
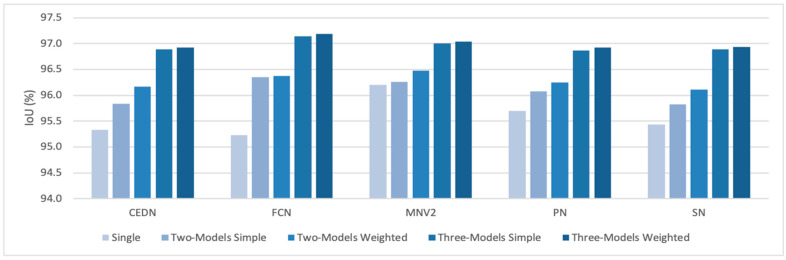
IoU comparison of single models and ensemble models.

From Table 3 and Table 4, the average of IoU, the average of IoU standard deviation and the average of the false prediction rate for individual models in both Two-Models ensemble and Three-Models ensemble were calculated. To calculate the average value of a single model, results are collected from Two-Models ensembles or Three-Models ensembles where the single model occurs. For example, the IoU of MNV2 in Two-Models ensemble is the average of IoU values taken from Two-Models ensembles where MNV2 occurs. Table 5 shows the IoU comparison for a single model without ensemble, the Two-Models ensemble using simple soft voting, the Two-Models ensemble using weighted soft voting, the Three-Models ensemble using simple soft voting and the Three-Models ensemble using weighted soft voting. It is observed that the MNV2 model, which has the highest IoU before ensemble, shows the highest IoU in Two-Models ensemble using weighted soft voting. The FCN model which has the lowest IoU before ensemble shows the highest IoU in the Two-Models ensemble using simple soft voting, as well as in the Three-Models ensemble. It shows the most improvement after ensemble, i.e., 95.2268% of IoU before ensemble is enhanced to 97.1839% after ensemble. The result shows that our approach to improve the accuracy does not degrade the accuracy of models with higher IoU, and at the same time improves the accuracy of models with lower IoU. Figure 5 is the graphical representation of IoU comparison of individual models for no ensemble, the Two-Models ensemble and the Three-Models ensemble. It is observed that the Three-Models ensemble shows the best result compared to no ensemble and the Two-Models ensemble, while the Two-Models ensemble shows a better result than no ensemble.

### 4.5. IoU Standard Deviation Comparison

Table 6 shows the comparison of IoU standard deviation. The IoU standard deviation is a measure of the amount of variation of IoU values. A low IoU standard deviation indicates that the IoU values tend to be close to the mean IoU. In Table 6, the experiment results of IoU standard deviation for the single model without ensemble, Two-Models ensemble using simple soft voting, Two-Models ensemble using weighted soft voting, Three-Models ensemble using simple soft voting and Three-Models ensemble using weighted soft voting are presented. The IoU standard deviation of the PN model, which is the lowest before ensemble, i.e., 3.7915, is reduced to 3.0581. The FCN model, which is the second highest of IoU standard deviation before ensemble, i.e., 5.6956, is reduced to 2.8583 after ensemble shows the most improvement. The result shows that the Two-Models and Three-Models ensemble produce a better result than the single model in most cases.

### 4.6. False Prediction Rate Comparison

Table 7 shows the experiment result of false prediction rate for the single model, Two-Models ensemble and Three-Models ensemble using the weighted soft voting method. In the table, the lower FDR + FNR value means the lower bias error as the bias error indicates the amount of difference between prediction and true values. The result shows that MNV2 has the lowest FDR + FNR value in the single model and Two-Models ensemble. MNV2, which is the lowest before ensemble, i.e., 3.8657, is reduced to 3.0057 after ensemble. The FCN model shows the most improvement after ensemble. The average of FDR + FNR shows that the Two-Models ensemble and Three-Models ensemble produce a lower bias error than the single model. It is also observed that the average of FDR + FNR is decreased as the number of single models combined for the ensemble is increased. In the table, |FDR-FNR| means the absolute value of FDR-FNR. This value indicates the amount of difference between FDR and FNR values. If this value is close to 0, then the model produces a well-balanced result in terms of falsely predicted regions. If the value is increased, then it means that falsely predicted positive regions are increased, while falsely predicted negative regions are decreased, or vice versa. From the average value in the table, it is observed that |FDR-FNR| values decrease significantly, as the number of models for the ensemble increases.

### 4.7. Examples of FNR Reduction

**Figure 6:**
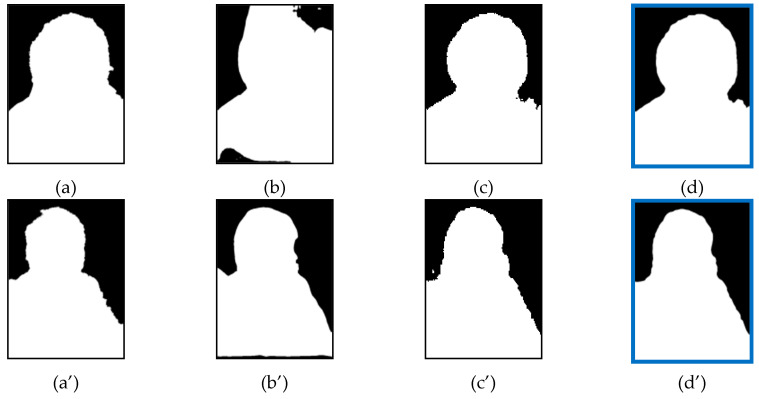
False Negative Rate (FNR) Reduction after Two-Models ensemble: (**a**) Ground Truth; (**b**) Fully Convolution Network (FCN) model (FNR = 3.4133); (**c**) MNV2 model (FNR = 2.9808); (**d**) FCN + MNV2 ensemble (FNR = 2.509); (**a**’) Ground Truth; (**b**’) FCN model (FNR = 2.0703); (**c**’) MNV2 model (FNR = 1.3135); (**d**’) FCN + MNV2 ensemble (FNR = 0.8886).

**Figure 7:**
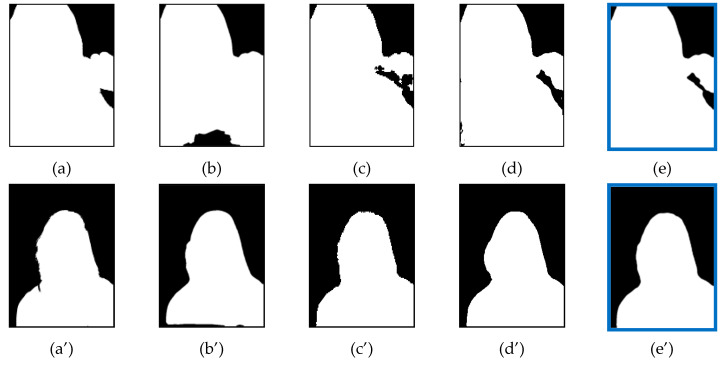
FNR Reduction after Three-Models ensemble: (**a**) Ground Truth; (**b**) FCN model (FNR = 4.1949); (**c**) MNV2 model (FNR = 3.1227); (**d**) SINet (SN) model (FNR = 1.9007); (**e**) FCN + MNV2 + SN ensemble (FNR = 1.2246); (**a**’) Ground Truth; (**b**’) FCN model (FNR = 2.0484); (**c**’) MNV2 model (FNR = 1.1793); (**d**’) SN model (FNR = 2.0181); (**e**’) FCN + MNV2 + SN ensemble (FNR = 0.8792).

Figure 6 and Figure 7 show the comparison of reduction in the FNR. The first picture shows ground truth image, the middle pictures show the segmented image masks of single models and the last picture shows the segmented image mask of the ensemble model. As the FNR indicates the error rate of missing regions compared to ground truth image, the result shows that the missing regions of single models were recovered after ensemble. In Figure 6, the examples show the reduction of FNR after the ensemble of FCN + MNV2. Figure 7 shows the reduction of FNR after the ensemble of FCN + MNV2 + SN.

### 4.8. Examples of FDR Reduction

**Figure 8:**
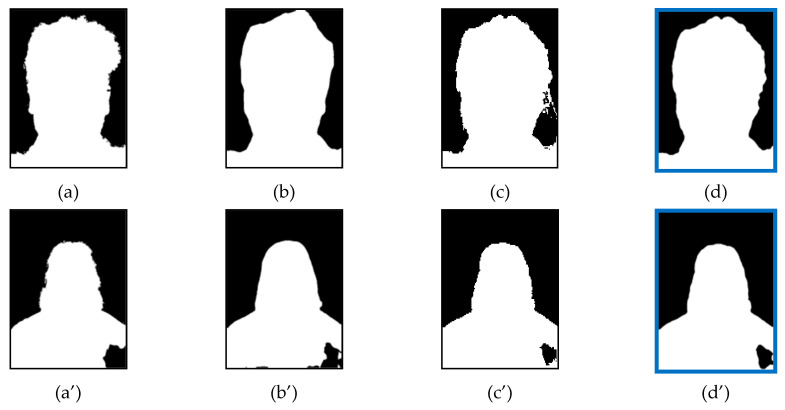
FDR Reduction after Two-Models ensemble: (**a**) Ground Truth; (**b**) FCN model (FDR = 4.1350); (**c**) MNV2 model (FDR = 4.7834); (**d**) FCN + MNV2 ensemble (FDR = 3.2252); (**a**’) Ground Truth; (**b**’) FCN model (FDR = 3.5817); (**c**’) MNV2 model (FDR = 3.007); (**d**’) FCN + MNV2 ensemble (FDR = 2.7056).

**Figure 9:**
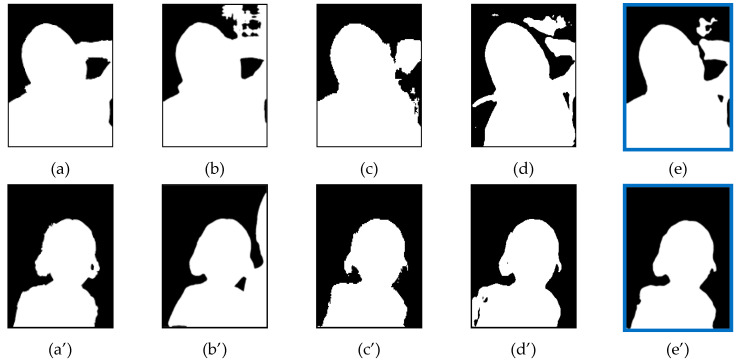
FDR Reduction after Three-Models ensemble: (**a**) Ground Truth; (**b**) FCN model (FNR = 10.3002); (**c**) MNV2 model (FDR = 3.4146); (**d**) SN model (FDR = 5.9966); (**e**) FCN + MNV2 + SN ensemble (FDR = 2.5271); (**a**’) Ground Truth; (**b**’) FCN model (FNR = 21.6792); (**c**’) MNV2 model (FDR = 2.4938); (**d**’) SN model (FDR = 5.2547); (**e**’) FCN + MNV2 + SN ensemble (FDR = 2.1429).

Figure 8 and Figure 9 show the comparison of reduction in the FDR. The first picture shows the ground truth image, while the middle pictures show the segmented image masks of single models and the last picture shows the segmented image mask of ensemble model. As the FDR indicates the error rate of extra regions compared to the ground truth image, the result shows that the extra regions of single models were removed after the ensemble. In Figure 8, the examples show the reduction of FDR after ensemble of FCN + MNV2. Figure 9 shows the reduction of FDR after ensemble of FCN + MNV2 + SN. The results show that the proposed ensemble approaches produces a lesser error rate than single models. This means that the proposed ensemble approaches produce better results than original single models.

### 4.9. Portrait Segmentation Examples

**Figure 10:**
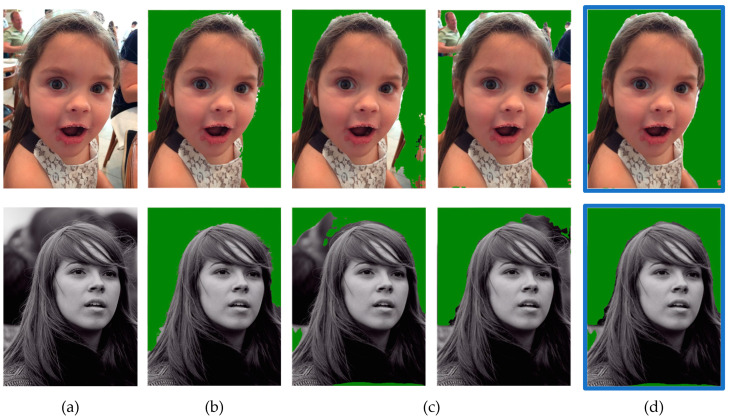
Segmented results of single models and Two-Models ensemble: (**a**) original image; (**b**) ground truth; (**c**) single models; and (**d**) proposed ensemble model.

**Figure 11:**
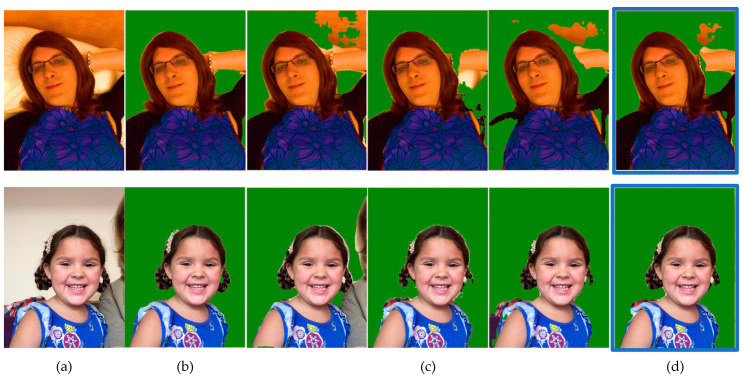
Segmented results of single models and Three-Models ensemble: (**a**) original image; (**b**) ground truth; (**c**) single models; and (**d**) proposed ensemble model.

Figure 10 and Figure 11 show the segmented results of single models and proposed ensemble models. The first column is the original image, the second column is ground truth segmentation, followed by single models’ segmentation, and the last column is the proposed ensemble model’s segmentation.

### 4.10. Cost Efficiency Analysis

In general, the ensemble of single classifiers can improve the accuracy of individual classifiers, but it requires more memory and computing power than single classifier, as the ensemble model has to execute all individual classifiers to combine its results. The total use of memory and computing power of ensemble model will be the summation of individual models included in it. The same results can be observed in our experiments. The averaged results of single models, Two-Models ensemble and Three-Models ensemble show that ensemble models generally improve segmentation accuracy, but also increase the use of memory and computing power.

To measure and compare the efficiency of single models and ensemble models, MER using Equation (10) and CER using Equation (11) were used. MER indicates the required memory size to achieve certain accuracy, where lower MER is more efficient in memory usage. CER indicates required computing power to achieve certain accuracy, while lower CER is more efficient in computing power usage. For the experiment, the EG1800 + CDI dataset was fed to the SN, PN, MNV3, MNV2 and FCN models, and the result of individual models was combined using the Two-Models ensemble and Three-Models ensemble. The simple soft voting method was used to make an ensemble of single models. Table 8, Table 9 and Table 10 show the efficiency rate of single models and ensemble models. The first column shows the model name, followed by the number of parameters (Params), FLOPs, IoU values, MER values and CER values of each models. The IoU value of ensemble models was calculated from the result of the simple soft voting ensemble.

In Table 8, the SN model shows the lowest MER and CER values compared to other single models. In the table, the SN, PN and MNV3 models show a lower MER and CER value than MNV2. This means that SN, PN and MNV3 are more cost efficient than MNV2, even though MNV2 shows the best IoU value, indicating the most accurate in segmentation. Table 9 shows the efficiency rate of the Two-Models ensemble. In the table, Params is the summation of Params of two individual models, FLOPs is the summation of FLOPs of two individual models and the IoU value is the result of the ensemble model. Comparing the FCN model in Table 8, the FCN + SN ensemble shows a lower MER and CER value, while the IoU value is higher than FCN single model, and it indicates that FCN + SN combination is a cost-efficient ensemble model with better accuracy than the FCN single model. Table 10 shows the efficiency rate of the Three-Models ensemble. In the table, Params is the summation of Params of three individual models, FLOPs is the summation of FLOPs of three individual models and the IoU value is the result of the ensemble model. Comparing the MNV2, which shows the highest accuracy among experimented single models, the MNV3 + PN + SN ensemble shows a lower MER and CER value, while the IoU value is higher than the MNV2 single model. The summation of Params and FLOPs of MNV3 + PN + SN are also less than the MNV2 single model. These results show that the ensemble model can perform with the same or higher accuracy than single models in a cost-efficient way.

## 5. Discussion

The analysis of experiment results shows that IoU, IoU standard deviation and false prediction rate of DL-based portrait segmentation models can be improved through an ensemble. This indicates that the accuracy of segmentation can be enhanced, and variance and bias errors can be reduced using an ensemble of DL-based portrait segmentation models. By increasing the number of single models participating in the ensemble from two to three, higher accuracy and lower prediction errors can be produced. The weighted soft voting method can be used to improve the accuracy of ensemble models that used the simple soft voting method.

The analysis of cost efficiency shows that the ensemble of DL-based models typically increases the use of memory and computing power, but also shows that the ensemble of DL-based models that perform more efficiently than a single DL-based model with higher accuracy using less memory and less computing power is possible.

## 6. Conclusions

In this paper, simple and efficient ensemble approaches for portrait segmentation using six state-of-the-art DL-based portrait segmentation models were proposed, and the experiment results of the Two-Models and Three-Models ensemble were presented and analyzed. The simple soft voting method and weighted soft voting methods as a Meta-Classifier in a stacking ensemble were used to combine the individual portrait segmentation models. The images from validation set of EG1800 and CDI dataset were used for the experiment. The IoU metric was used to evaluate the accuracy of single models and the proposed ensemble approach. The IoU standard deviation and false prediction rate were analyzed to evaluate the improvement in variance and bias errors. The efficiency rate was analyzed to evaluate the efficiency of single models and the proposed ensemble approach. The experiment result showed that the IoU value, IoU standard deviation and false prediction rate of single models were significantly improved after ensemble. The result of the ensemble using the weighted soft voting method showed better accuracy than the simple soft voting method. It was also observed that the Three-Models ensemble showed better results than the Two-Models ensemble in terms of accuracy, variance and bias errors. The analysis of cost efficiency showed that the ensemble of DL-based portrait segmentation models typically increased the use of memory and computing power. However, it has also shown that the ensemble of DL-based portrait segmentation models can perform more efficiently than a single DL-based portrait segmentation model with higher accuracy, using less memory and less computing power.

In this paper, we evaluated six state-of-the-art DL-based portrait segmentation models and compared them with the proposed ensemble approach. We also introduced methods to improve the performance of DL-based portrait segmentation models using the ensemble approach, as well as methods to evaluate the performance of DL-based portrait segmentation models and its ensemble models. We hope these findings will benefit other researchers.

## Figures and Tables

**Table 1:** Training and testing dataset of models.

Model	Training Dataset	Resolution	Testing Dataset
CEDN [38]	AISegment	224 × 224	EG1800 + CDI
FCN [35]	PASCAL VOC 2011	500 × 500	EG1800 + CDI
MobileNetV2 [36]	Custom	128 × 128	EG1800 + CDI
MobileNetV3 [37]	Custom	224 × 224	EG1800 + CDI
PortraitNet [33]	EG1800	224 × 224	EG1800 + CDI
SINet [34]	EG1800	224 × 224	EG1800 + CDI

**Table 2:** Result of single portrait segmentation models.

Model	IoU (%)	IoU Std. (%)	FNR (%)	FDR (%)	FNR + FDR (%)
MNV2	**96.2082**	3.9133	1.8405	2.0252	**3.8657**
PN	95.6992	**3.7915**	2.6215	1.7656	4.3871
SN	95.4326	4.4658	3.1167	**1.5555**	4.6722
CEDN	95.3317	5.724	2.9089	1.8587	4.7676
FCN	95.2268	5.6956	**1.1538**	3.6887	4.8425

Note: The best results are highlighted in bold.

**Table 3:** Result of Two-Models ensemble.

Two-Models	Simple Soft Voting			Weighted Soft Voting		
	IoU (%)	IoU Std. (%)	FNR + FDR (%)	IoU (%)	IoU Std. (%)	FNR + FDR (%)
CEDN + FCN	96.1564	4.2910	3.9039	96.1761	4.2368	3.8844
CEDN + MNV2	96.0816	4.5499	3.9821	96.5388	3.8342	3.5260
CEDN + PN	95.6933	4.4740	4.3710	96.0668	3.7005	4.0086
CEDN + SN	95.4314	5.0148	4.6411	95.8786	4.2163	4.2067
FCN + MNV2	96.5291	3.2504	3.5288	96.5343	**3.2250**	3.5234
FCN + PN	**96.5817**	**3.2392**	**3.4697**	**96.6145**	3.2251	**3.4368**
FCN + SN	96.1484	3.9143	3.9193	96.1824	3.8603	3.8850
MNV2 + PN	96.3509	3.7448	3.7013	96.3666	3.7380	3.6855
MNV2 + SN	96.0737	4.2989	3.9832	96.4589	3.7204	3.6055
PN + SN	95.6686	4.1997	4.4049	95.9469	3.7007	4.1324

Note: The best results are highlighted in bold.

**Table 4:** Result of Three-Models ensemble.

Three-Models	Simple Soft Voting			Weighted Soft Voting		
	IoU (%)	IoU Std. (%)	FNR + FDR (%)	IoU (%)	IoU Std. (%)	FNR + FDR (%)
CEDN + FCN + MNV2	97.2154	2.9288	2.8215	97.2158	2.9403	2.8207
CEDN + FCN + PN	97.0443	3.1649	2.9962	97.1085	3.1375	2.9284
CEDN + FCN + SN	97.0900	2.9497	2.9502	97.1310	2.8957	2.9060
CEDN + MNV2 + PN	96.7472	3.2006	3.3030	96.7952	3.2640	3.2563
CEDN + MNV2 + SN	96.7973	3.1886	3.2523	96.8070	3.2077	3.2440
CEDN + PN + SN	96.4518	3.2560	3.6061	96.4617	3.3474	3.5954
FCN + MNV2 + PN	97.2357	2.7784	2.7979	97.2508	2.7654	2.7815
FCN + MNV2 + SN	**97.3061**	**2.5434**	**2.7305**	**97.3132**	**2.5360**	**2.7224**
FCN + PN + SN	96.9507	2.9330	3.0917	97.0841	2.8749	2.9526
MNV2 + PN + SN	96.7476	2.9922	3.3023	96.8395	2.9594	3.2090

Note: The best results are highlighted in bold.

**Table 5:** Intersection over Union (IoU) value comparison.

Model	Without Ensemble (%)	Two-Models Ensemble		Three-Models Ensemble	
		Simple Soft Voting (%)	Weighted Soft Voting (%)	Simple Soft Voting (%)	Weighted Soft Voting (%)
CEDN	95.3317	95.8407	96.1651	96.8910	96.9199
FCN	95.2268	**96.3539**	96.3768	**97.1404**	**97.1839**
MNV2	**96.2082**	96.2588	**96.4747**	97.0082	97.0369
PN	95.6992	96.0736	96.2487	96.8629	96.9233
SN	95.4326	95.8305	96.1167	96.8906	96.9394

Note: The best results are highlighted in bold.

**Table 6:** IoU standard deviation comparison.

Model	Without Ensemble (%)	Two-Models Ensemble		Three-Models Ensemble	
		Simple Soft Voting (%)	Weighted Soft Voting (%)	Simple Soft Voting (%)	Weighted Soft Voting (%)
CEDN	5.7240	4.5824	3.9970	3.1148	3.1321
FCN	5.6956	**3.6737**	3.6368	**2.8830**	**2.8583**
MNV2	3.9133	3.9610	**3.6294**	2.9387	2.9455
PN	**3.7915**	3.9144	3.5911	3.0542	3.0581
SN	4.4658	4.3569	3.8744	2.9772	2.9702

Note: The best results are highlighted in bold.

**Table 7:** False Prediction Rate comparison.

Model	No Ensemble		Two-Models Ensemble		Three-Models Ensemble	
	FDR + FNR (%)	|FDR-FNR| (%)	FDR + FNR (%)	|FDR-FNR| (%)	FDR + FNR (%)	|FDR-FNR| (%)
CEDN	4.7676	1.0502	3.9064	0.8293	3.1251	**0.0074**
FCN	4.8425	2.5349	3.6824	0.6087	**2.8519**	0.5696
MNV2	**3.8657**	**0.1847**	**3.5851**	**0.4701**	3.0057	0.0913
PN	4.3871	0.8559	3.8158	0.9000	3.1205	0.0530
SN	4.6722	1.5612	3.9574	0.8845	3.1049	0.0167
Average	4.5070	1.2374	3.7894	0.7385	3.0416	0.1476

Note: The best results are highlighted in bold.

**Table 8:** Efficiency rate of single models.

Model	Params (M)	FLOPs (G)	IoU (%)	MER	CER
SN	0.087	0.15	95.4326	0.091	0.157
PN	2.115	0.209	95.6992	2.210	0.218
MNV3	1.192	2.3724	94.7597	1.258	2.504
MNV2	3.625	7.227	96.2082	3.768	7.512
FCN	134.27	62.89	95.2268	141.000	66.042
Average	28.2578	14.5697	95.4653	29.6654	15.2866

**Table 9:** Efficiency rate of Two-Models ensemble.

Model	Params (M)	FLOPs (G)	IoU (%)	MER	CER
PN + SN	2.202	0.359	95.6686	2.302	0.375
MNV3 + SN	1.279	2.5224	95.6836	1.337	2.636
MNV3 + PN	3.307	2.5814	95.9492	3.447	2.690
MNV2 + SN	3.712	7.377	96.0737	3.864	7.678
MNV2 + PN	5.74	7.436	96.3509	5.957	7.718
MNV3 + MNV2	4.817	9.5994	96.0535	5.015	9.994
FCN + PN	136.385	63.099	96.5817	141.212	65.332
FCN + SN	134.357	63.04	*96.1484*	*139.739*	*65.565*
MNV3 + FCN	135.462	65.2624	96.016	141.083	67.970
FCN + MNV2	137.895	70.117	96.5291	142.853	72.638
Average	56.516	29.1394	96.1055	58.681	30.260

**Table 10:** Efficiency rate of Three-Models ensemble.

Model	Params (M)	FLOPs (G)	IoU (%)	MER	CER
MNV3 + PN + SN	3.394	2.7314	96.5053	3.517	2.830
MNV2 + PN + SN	5.827	7.586	96.7476	6.023	7.841
MNV3 + MNV2 + SN	4.904	9.7494	96.6016	5.077	10.092
MNV3 + MNV2 + PN	6.932	9.8084	96.5125	7.182	10.163
FCN + PN + SN	136.472	63.249	96.9507	140.764	65.238
MNV3 + FCN + SN	135.549	65.4124	96.9736	139.779	67.454
MNV3 + FCN + PN	137.577	65.4714	96.8928	141.989	67.571
FCN + MNV2 + SN	137.982	70.267	97.3061	141.802	72.212
FCN + MNV2 + PN	140.01	70.326	97.2357	143.990	72.325
MNV3 + FCN + MNV2	139.087	72.4894	96.7122	143.815	74.954
Average	84.773	43.7090	96.8438	87.394	45.068

## Data Availability

Data available in a publicly accessible repository. The data presented in this study are openly available in reference [43,44].

## References

[1] Guo Y., Liu Y., Georgiou T., Lew M.S. (2018). A review of semantic segmentation using deep neural networks. Int. J. Multimed. Inf. Retr..

[2] Haralick R.M., Shapiro L.G. (1985). Image segmentation techniques. Comput. Vis. Graph. Image Process..

[3] Minaee S., Boykov Y., Porikli F., Plaza A., Kehtarnavaz N., Terzopoulos D. (2020). Image Segmentation Using Deep Learning: A Survey. arXiv.

[4] Aly A.A., Deris S.B., Zaki N. (2011). Research Review For Digital Image Segmentation Techniques. International Journal of Computer Science & Information Technology. Int. J. Comput. Sci. Inform. Technol..

[5] Khan M.W. (2014). A survey: Image segmentation techniques. Int. J. Future Comput. Commun..

[6] Vantaram S.R., Saber E. (2012). Survey of contemporary trends in color image segmentation. J. Electron. Imaging.

[7] Zuva T., Olugbara O.O., Ojo S.O., Ngwira S.M. (2011). Image segmentation, available techniques, developments and open issues. Can. J. Image. Proc. Comput. Vis..

[8] Yu P., Qin A.K., Clausi D.A. (2012). Unsupervised polarimetric SAR image segmentation and classification using region growing with edge penalty. IEEE Trans. Geosci. Remote Sens..

[9] Patil D.D., Deore S.G. (2013). Medical image segmentation: A review. Int. J. Comput. Sci. Mob. Comput..

[10] Arbelaez P., Maire M., Fowlkes C., Malik J. (2011). Contour detection and hierarchical image segmentation. IEEE Trans. Pattern. Anal. Mach. Intell..

[11] Geman S. (1984). Stochastic relaxation, Gibbs distribution, and Bayesian restoration of images. IEEE Trans. Pattern. Anal. Mach. Intell..

[12] Muthukrishnan R., Radha M. (2011). Edge detection techniques for image segmentation. Int. J. Comput. Sci. Inform. Technol..

[13] Milan S., Vaclav H., Roger B. (2013). Image Processing, Analysis, and Machine Vision.

[14] Garcia-Garcia A., Orts-Escolano S., Oprea S., Villena-Martinez V., Garcia-Rodriguez J. (2017). A Review on Deep Learning Techniques Applied to Semantic Segmentation. arXiv.

[15] Miao J., Sun K., Liao X., Leng L., Chu J. (2020). Human Segmentation Based on Compressed Deep Convolutional Neural Network. IEEE Access.

[16] Zhao B., Feng J., Wu X., Yan S. (2017). A survey on deep learning-based fine-grained object classification and semantic segmentation. Int. J. Autom. Comput..

[17] Rokach L. (2010). Ensemble-based classifiers. Artif. Intell. Rev..

[18] Goodfellow I., Bengio Y., Courville A. (2016). Deep Learning.

[19] Breiman L. (1996). Stacked regressions. Mach. Learn..

[20] Warfield S.K., Zou K.H., Wells W.M. (2004). Simultaneous Truth and Performance Level Estimation (STAPLE): An algorithm for the Vaidation of Image Segmentation. IEEE Trans. Med. Imaging.

[21] Rohlfing T., Maurer C.R., Maurer C.R. (2005). Shape-based averaging for combination of multiple segmentations. Med. Image Comput. Comput. Interv..

[22] Hansen L.K., Salamon P. (1990). Neural Network Ensembles. IEEE Trans. Pattern Anal. Mach. Intell..

[23] Singh V., Mukherjee L., Peng J., Xu J. (2010). Ensemble clustering using semidefinite programming with applications. Mach. Learn..

[24] Zuo Y., Drummond T. Fast Residual Forests: Rapid Ensemble Learning for Semantic Segmentation. Proceedings of the 1st Annual Conference on Robot Learning (CoRL 2017).

[25] Holliday A., Barekatain M., Laurmaa J., Kandaswamy C., Prendinger H. (2017). Speedup of deep learning ensembles for semantic segmentation using a model compression technique. Comput. Vis. Image Underst..

[26] Nigam I., Huang C., Ramanan D. Ensemble Knowledge Transfer for Semantic Segmentation. Proceedings of the 2018 IEEE Winter Conference on Applications of Computer Vision (WACV).

[27] Marmanis D., Wegner J.D., Galliani S., Schindler K., Datcu M., Stilla U. (2016). Semantic Segmentation of Aerial Images with an Ensemble of Cnns. ISPRS Ann. Photogramm. Remote Sens. Spat. Inf. Sci..

[28] Koren Y. (2009). The BellKor Solution to the Netflix Grand Prize. https://netflixprize.com/assets/GrandPrize2009_BPC_BellKor.pdf.

[29] Kim Y.W., Rose J.I., Krishna A.V.N. Accuracy Enhancement of Portrait Segmentation by Ensembling Deep Learning Models. Proceedings of the 2020 Fifth International Conference on Research in Computational Intelligence and Communication Net-works (ICRCICN).

[30] Liu X., Deng Z., Yang Y. (2019). Recent progress in semantic image segmentation. Artif. Intell. Rev..

[31] Du X., Wang X., Li D., Zhu J., Tasci S., Upright C., Walsh S., Davis L. Boundary-sensitive network for portrait segmentation. Proceedings of the 2019 14th IEEE International Conference on Automatic Face & Gesture Recognition.

[32] Garcia-Garcia A., Orts-Escolano S., Oprea S., Villena-Martinez V., Martinez-Gonzalez P., Garcia-Rodriguez J. (2018). A survey on deep learning techniques for image and video semantic segmentation. Appl. Soft Comput..

[33] Zhang S.H., Dong X., Li H., Li R., Yang Y.L. (2019). PortraitNet: Real-time portrait segmentation network for mobile device. Comput. Graph..

[34] Park H., Sjösund L.L., Yoo Y., Monet N., Bang J., Kwak N. SINet: Extreme Lightweight Portrait Segmentation Networks with Spatial Squeeze Modules and Information Blocking Decoder. Proceedings of the 2020 IEEE Winter Conference on Applications of Computer Vision (WACV).

[35] Shelhamer E., Long J., Darrell T. (2015). Fully Convolutional Networks for Semantic Segmentation. IEEE Trans. Pattern Anal. Mach. Intell..

[36] Sandler M., Howard A., Zhu M., Zhmoginov A., Chen L.C. MobileNetV2: Inverted Residuals and Linear Bottlenecks. Proceedings of the 2018 IEEE/CVF Conference on Computer Vision and Pattern Recognition.

[37] Howard A., Sandler M., Chu G., Chen L.-C., Chen B., Tan M., Wang W., Zhu Y., Pang R., Vasudevan V. Searching for mobilenetv3. Proceedings of the IEEE/CVF International Conference on Computer Vision.

[38] Yang J., Price B., Cohen S., Lee H., Yang M.H. Object contour detection with a fully convolutional encoder-decoder network. Proceedings of the 29th IEEE Conference on Computer Vision and Pattern Recognition.

[39] Park H., Sjösund L.L., Yoo Y., Bang J., Kwak N. (2019). ExtremeC3Net: Extreme Lightweight Portrait Segmentation Networks Using Advanced C3-Modules. arXiv.

[40] Chen L.-C., Papandreou G., Schroff F., Adam H. (2017). Rethinking Atrous Convolution for Semantic Image Segmentation. arXiv.

[41] Chen X., Qi D., Shen J. (2019). Boundary-Aware Network for Fast and High-Accuracy Portrait Segmentation. arXiv.

[42] Zhou Z.-H. (2012). Ensemble Methods, Foundations and Algorithms.

[43] AISegment.com—Matting Human Datasets|Kaggle. https://www.kaggle.com/laurentmih/aisegmentcom-matting-human-datasets.

[44] EG1800.zip_Free High-Speed Download|Baidu Netdisk-Share Unlimited. https://pan.baidu.com/s/1myEBdEmGz6ufniU3i1e6Uw.

[45] Wolpert D.H. (1992). Stacked generalization. Neural Netw..

[46] Smyth P., Wolpert D. (1999). Linearly combining density estimators via stacking. Mach. Learn..

[47] Talbi F., Alim F., Seddiki S., Mezzah I., Hachemi B. Separable convolution Gaussian smoothing filters on a xilinx FPGA platform. Proceedings of the Fifth International Conference on the Innovative Computing Technology (INTECH 2015).

[48] Kabbai L., Sghaier A., Douik A., Machhout M. (2016). FPGA implementation of filtered image using 2D Gaussian filter. Int. J. Adv. Comput. Sci. Appl..

